# Inhalational exposure to dimethyl sulfate vapor followed by reactive airway dysfunction syndrome

**DOI:** 10.4103/0019-5278.75700

**Published:** 2010

**Authors:** Abbas Aghabiklooei, Nasim Zamani, Hamidreza Shiva, Nader Rezaei

**Affiliations:** Department of Forensic Medicine and Clinical Toxicology, Firouzgar Teaching Hospital, Tehran University of Medical Sciences, Tehran, Iran; 1 Department of Forensic Medicine and Clinical Toxicology, Tehran University of Medical Sciences, Tehran, Iran; 2 Department of Emergency Medicine, Tehran University of Medical Sciences, Tehran, Iran; 3 Department of Internal Medicine Ward, Firouzgar Hospital, Tehran University of Medical Sciences, Tehran, Iran

**Keywords:** Dimethyl sulfate, reactive airway dysfunction syndrome, poisoning, vapor exposure

## Abstract

Dimethyl sulfate (DMS) is an oily liquid used as a solvent, stabilizer, sulfonation agent, and catalyst. Exposure to DMS primarily happens in the workplace via inhalational contact and damages the upper and lower airways. Our manuscript reports a case of DMS-related reactive airway dysfunction syndrome (RADS). The patient was a healthy 29-year-old man who was referred to our ER after accidental exposure to the vapor of DMS with the complaint of dyspnea, dry cough, photophobia, and hoarseness. His vital signs were normal except for a low-grade fever. Redness of the pharynx, conjunctivitis, and cholinergic signs and symptoms were present. Conservative management with O_2_ and fluid therapy was initiated. Twenty hours later, the patient became drowsy and his respiratory symptoms exacerbated; chest X-ray revealed haziness in the base of the right lung and prominence of the vessels of the lung hillum. After 1 week, the liver transaminases rose and C-reactive protein elevated (2+). The patient got better with conservative treatment and was discharged after 9 days; however, exertional dyspnea, wheezing, and thick white sputum persisted and therefore, reactive airway dysfunction syndrome (RADS) related to DMS vapor was confirmed which was treated by prednisolone. Exertional dyspnea continued up to 10 months. Hoarseness lasted for 6 months. This case shows that DMS vapor inhalation can cause RADS especially in the chemical workers who continue working in the contaminated place despite the relatively good air conditioning.

## INTRODUCTION

Dimethyl sulfate (DMS) is a colorless, onion-like odorous oily liquid used as a methylation agent in organic synthesis. It is also used as a solvent, stabilizer, sulfonation agent, and catalyst. The end applications of DMS include surfactants, pesticides, water treatment chemicals, dyes, perfumes, flavors, pharmaceuticals, and rubber and photographic chemicals. It has previously been used as a war gas.[1] It may easily vaporize at room temperature and therefore, exposure to DMS primarily happens in the workplace via inhalation or dermal contact.[2] Acute inhalation exposure primarily damages the upper and lower airways and may be short- or long-term. There were scant articles on human DMS poisoning and to our best knowledge, no article has reported DMS-related reactive airway dysfunction syndrome (RADS).

## CASE REPORT

**Figure 1:**
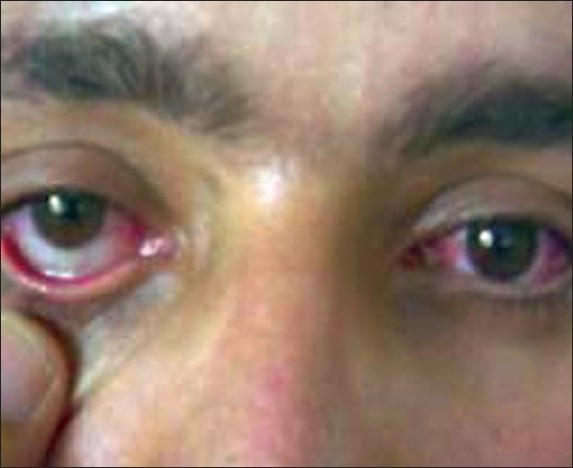
Conjunctivitis

Our patient was a healthy 29-year-old man who was referred to the emergency department of Firouzgar hospital after accidental exposure to the vapor of DMS while transporting a broken bottle containing it. The patient had opened the windows but had continued working in the same place for another 6 h. He did not mention any history of atopy. At presentation, he was completely awake with the complaint of dyspnea, dry cough, photophobia, and hoarseness. His blood pressure, pulse rate, respiratory rate, and oral temperature were 115/80 mmHg, 94 b/min, 28/min, and 38 °C, respectively. Redness of the pharynx, conjunctivitis [Figure 1], and cholinergic signs and symptoms (including miosis, diaphoresis, coryza, and lacrimation) were evident. In physical examination, generalized rhonchi and wheezing were auscultated all over the lungs. Uvula was very large and elongated and its color had changed to white. Severe edema and pallor of the soft palate and larynx was obvious. In the first ABG, o_2_ saturation was 88%, PaO_2_ was 52 mmHg, and Pco_2_ was 30.1 mmHg. Hypoxia responded to nasal O_2_ administration. Chest X-ray was normal. In indirect laryngoscopy, mild swelling of the glottis was documented and hydrocortisone was started. Atropine was administered to relieve cholinergic signs and symptoms and conservative management with O_2_ and fluid therapy was initiated. Blood urea nitrogen, creatinine, sodium, potassium, calcium, alkaline phosphatase, SGOT, and SGPT levels were 15, 1, 144, 3.6, 8.9, 207, 27, and 20, respectively. After 20 h, the patient became drowsy and his respiratory symptoms exacerbated and second chest X-ray revealed haziness in the base of the right lung and prominence of the vessels of the lung hillum. Therefore, the patient was admitted to the intensive care unit.

**Figure 2:**
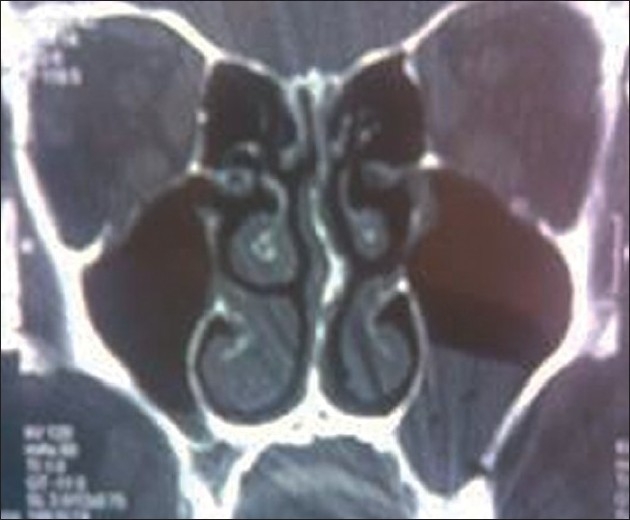
Left maxillary sinus accumulation in coronal CT

On the second day of admission, exertional dyspnea and hoarseness were more prominent. The patient had low-grade fever, diffuse bilateral coarse crackles, tachycardia, and burn sensation at mouth, throat, and retro-sternal region. Coronal CT scan without contrast revealed accumulation of fluid in left maxilar sinus [Figure 2]. In the CXR of the second day, paracardiac haziness was evident and therefore, intravenous ceftriaxone was initiated.

On the 3^rd^ day, CP angle was blunted in CXR and leukocytosis appeared. Sonography detected little fluid in the right pleural space. Pleural tap under the guidance of the ultrasonography was tried which was unsuccessful.

The patient was better on the 4^th^ day, and therefore, he was discharged from the ICU and admitted to the ward. However, his hoarseness and conjunctivitis persisted.

**Figure 3:**
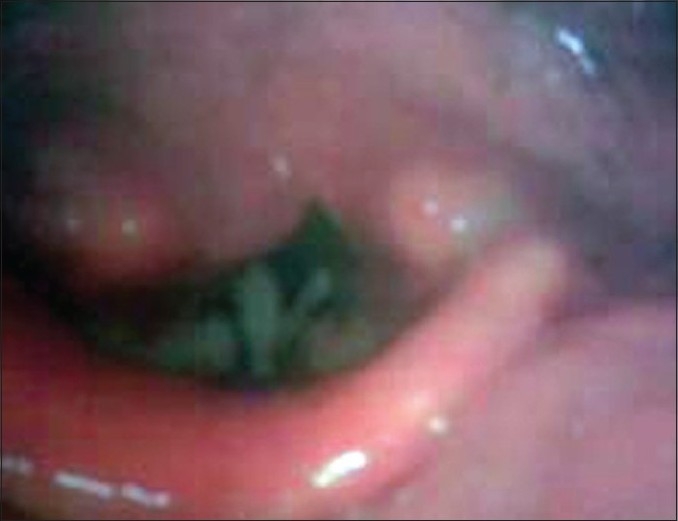
Laryngeal edema in stroboscopy

After 1 week, the liver transaminases rose (SGOT=73; SGPT = 214) and C-reactive protein elevated (2+). Although congestion and hyperemia of the conjunctives was evident, in ophthalmologic consultation, no corneal abrasion was detected. Supportive care focused on airway management; the patient relatively improved with conservative therapy and was discharged after 9 days. He was followed weekly for 1 month and monthly, afterwards. One month after discharge, high resolution computed tomography and pulmonary function tests were performed and were both normal; however, exertional dyspnea, wheezing, and tick white sputum persisted and therefore, reactive airway dysfunction syndrome (RADS) related to DMS vapor was confirmed; thus, prednisolone was continued for 6 months. Exertional dyspnea continued up to 10 months. Hoarseness lasted for 6 months. In stroboscopy [Figure 3], laryngeal edema was still obvious after 6 months.

## DISCUSSION

Reactive airway dysfunction syndrome is an occupational asthma characterized by immediate onset of asthma after a single exposure to irritating vapor, fume, or smoke. The criteria for diagnosis of RADS include absence of previous respiratory complaints, occurrence of the symptoms after a single exposure, positive history of exposure to an irritant gas, smoke, fume or vapor in high concentrations, occurrence of the symptoms within 24 h of exposure and persistence of them for a minimum of 3 months, simulation of the symptoms of asthma (cough, wheezing, and dyspnea), positive methacholine challenge test, and ruled out other types of pulmonary disease.[3,4]

Chemical agents with a known effect to cause RADS include ammonia, bleaching agents, chlorine, cleaning agents, detergents, disinfectants, formaldehyde, hydrochloric acid, pesticides, polyurethane, resins/fluoro-resins, sealers, sodium hydroxide, sulfur dioxide, sulfuric acid, and waterproofing agents. However, no study was found to report DMS-related RADS.

Alteration of the receptor thresholds in the airways caused by extensive inflammation associated with short-term exposure which results in non-specific bronchial hyper-reactivity and direct damage to the bronchial mucosa altering smooth muscle responsiveness are main suggested hypotheses for RADS.[5]

Most of the articles suggest that RADS usually occur after exposure to high concentration of DMS vapor, the higher is the concentration of the inhaled agent, the greater is the likelihood of occurrence of the symptoms of asthma.[3] However, setting exposure limits, below which exposures can be called ’’safe,’’ is difficult.[6] In our case, although the concentration of DMS had decreased due to the opening of the windows, the symptoms developed because of the prolonged contact period with the vapor. On the other hand, it seems that since the floor decontamination had not been performed, DMS continued evaporate in the room temperature and this was the cause of poisoning in our case.

Such as our case, inflammation of eyes, nose, oropharynx, and airways are initial signs of acute toxicity.[7] Severe airway edema, necrosis, and non-cardiogenic pulmonary edema also have been reported. Convulsion, delirium, coma, and renal, hepatic, and cardiac failure may also occur.[7] In our case, after one week, the liver transaminases rose, and therefore, intravenous *N*-acetylcysteine was administered and enzymes returned to normal range after two weeks.

The diagnosis is based on history of exposure, clinical signs and symptoms, and results of respiratory function tests and bronchoscopy.[8] Rippy’s study showed that delayed toxicity with DMS can occur prior to any warning symptoms. Symptoms may be delayed to 6–24 h. Delayed presentation of symptoms may permit unnoticed exposure to lethal quantities of DMS.[7] In our case, these symptoms became more obvious after 20 h, as well.

In highly exposed rescue workers at the World Trade Center, bronchial hyper-reactivity at 1 and 3 months post-exposure was the only significant predictor for the development of RADS. Treatment with corticosteroids is advocated in these patients.[5] Our case received prednisolone for 6 months to regain his acceptable pulmonary function. Serial monitoring of bronchial hyper-reactivity is often advocated. Tapering of the inhaled corticosteroids is performed based on the clinical response to the treatment. The response to treatment is variable and the complete resolution of the condition may take months or years to happen.[5] Complete improvement in our patient’s respiratory function took 1 year to happen.

## CONCLUSION

This case report shows that DMS vapor inhalation can cause RADS especially in the chemical workers who continue working in the contaminated place despite the relatively good air conditioning. The victim must immediately be removed from the exposure site to fresh air and contaminated workplace should be cleaned up by removing the material by vacuum sweeper with disposal container to minimize dust generation.
